# Inhibition of Bruton tyrosine kinase in patients with severe COVID-19

**DOI:** 10.1126/sciimmunol.abd0110

**Published:** 2020-06-05

**Authors:** Mark Roschewski, Michail S. Lionakis, Jeff P. Sharman, Joseph Roswarski, Andre Goy, M. Andrew Monticelli, Michael Roshon, Stephen H. Wrzesinski, Jigar V. Desai, Marissa A. Zarakas, Jacob Collen, Keith Rose, Ahmed Hamdy, Raquel Izumi, George W. Wright, Kevin K. Chung, Jose Baselga, Louis M. Staudt, Wyndham H. Wilson

**Affiliations:** ^1^Lymphoid Malignancies Branch, National Cancer Institute, Bethesda, MD; ^2^Fungal Pathogenesis Section_,_ Laboratory of Clinical Immunology and Microbiology, National Institute of Allergy and Infectious Diseases, Bethesda, MD; ^3^Willamette Valley Cancer Institute and Research Center, US Oncology, Eugene, OR; ^4^Hematology-Oncology Department, Walter Reed National Military Medical Center, Bethesda, MD; ^5^John Theurer Cancer Center, Hackensack Meridian and School of Medicine at Seton Hall, NJ; ^6^Rocky Mountain Cancer Center, US Oncology, Colorado Springs, CO; ^7^Department of Emergency Medicine, Penrose-St. Francis Health Services, Colorado Springs, CO; US Acute Care Solutions, Canton, OH; ^8^Department of Medicine, St. Peter’s Hospital and US Oncology, Albany, NY; ^9^Department of Medicine, Uniformed Services University of the Health Sciences, Bethesda, MD; ^10^Acerta Pharma, South San Francisco, CA; ^11^Biometric Research Branch, Division of Cancer Diagnosis and Treatment, National Cancer Institute, Bethesda, MD, USA ^12^AstraZeneca, One MedImmune Way, Gaithersburg, MD

## Abstract

Patients with severe COVID-19 have a hyperinflammatory immune response suggestive of macrophage activation. Bruton tyrosine kinase (BTK) regulates macrophage signaling and activation. Acalabrutinib, a selective BTK inhibitor, was administered off-label to 19 patients hospitalized with severe COVID-19 (11 on supplemental oxygen; 8 on mechanical ventilation), 18 of whom had increasing oxygen requirements at baseline. Over a 10-14 day treatment course, acalabrutinib improved oxygenation in a majority of patients, often within 1-3 days, and had no discernable toxicity. Measures of inflammation – C-reactive protein and IL-6 – normalized quickly in most patients, as did lymphopenia, in correlation with improved oxygenation. At the end of acalabrutinib treatment, 8/11 (72.7%) patients in the supplemental oxygen cohort had been discharged on room air, and 4/8 (50%) patients in the mechanical ventilation cohort had been successfully extubated, with 2/8 (25%) discharged on room air. Ex vivo analysis revealed significantly elevated BTK activity, as evidenced by autophosphorylation, and increased IL-6 production in blood monocytes from patients with severe COVID-19 compared with blood monocytes from healthy volunteers. These results suggest that targeting excessive host inflammation with a BTK inhibitor is a therapeutic strategy in severe COVID-19 and has led to a confirmatory international prospective randomized controlled clinical trial.

## Teaser

Acalabrutinib targets activated BTK in macrophages and was associated with reduced inflammation and clinical improvement in COVID-19.

## INTRODUCTION

Coronavirus 2019 (COVID-19) is a new pandemic disease caused by a single-stranded RNA zoonotic virus termed severe acute respiratory syndrome coronavirus 2 (SARS-CoV-2) (*1*). The spectrum of COVID-19 ranges from a mild respiratory illness to a severe disease requiring hospitalization in up to a third of patients, with frequent progression to acute respiratory distress syndrome (ARDS) and a high mortality (*2*). It has been reported that COVID-19 patients can have a biphasic clinical course with deterioration following initial improvement, consistent with a delayed and exaggerated immune activation (*2*–*4*). A virus-induced hyperinflammatory response or “cytokine storm” (*5*) has been hypothesized to be a major pathogenic mechanism of ARDS in these patients through modulation of pulmonary macrophages, dendritic cells and/or neutrophils (*6*–*10*). Indeed, patients with COVID-19 have elevated blood levels of multiple inflammatory cytokines and chemokines (IL-1β, IL-6, IL-7, IL-8, IL-9, IL-10, G-CSF, GM-CSF, IFN-γ, IP-10, MCP-1, and MIP-1α), and those requiring admittance to an intensive care unit have even higher levels of many of these (*11*, *12*). The hyperinflammatory response in COVID-19 shares biological characteristics with macrophage activation syndrome, suggesting that targeting the innate immune system may be an effective strategy (*13*).

**Fig. 1:**
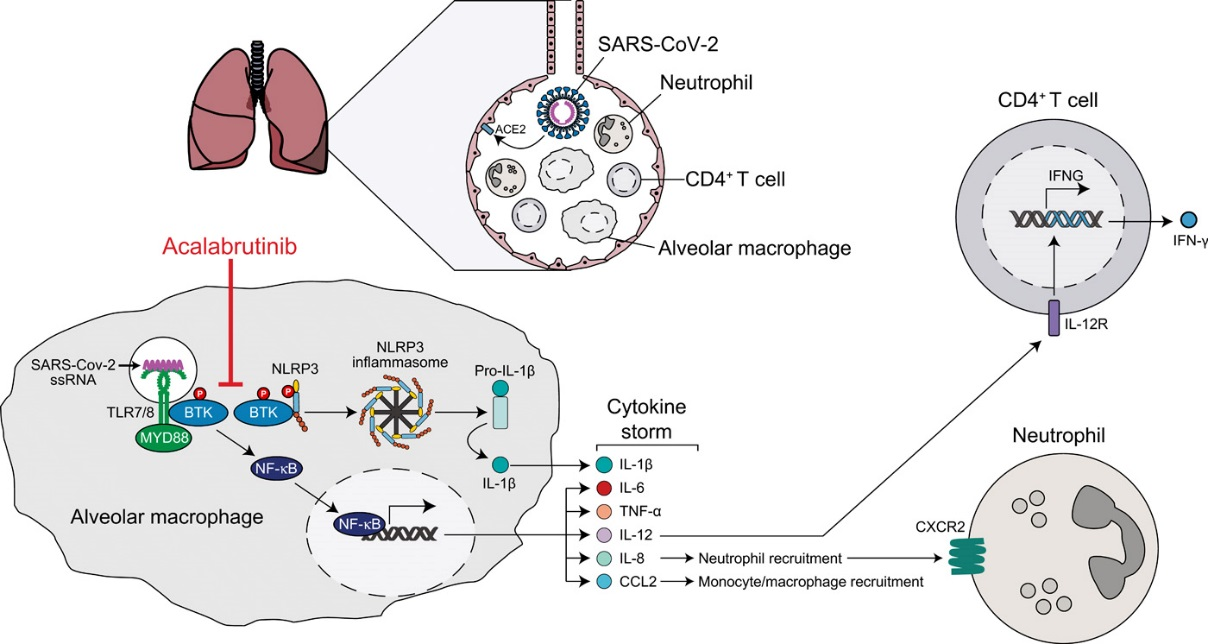
Model of BTK-dependent hyper-inflammation in severe COVID-19. Binding of SARS-CoV2 to ACE2 on respiratory epithelia initiates infection. Hypothetically, macrophages may participate in the COVID-19 inflammatory response by phagocytic uptake of viral particles or cellular debris containing viral single-stranded RNA (ssRNA). ssRNA can bind to TLR7 and TLR8, thereby recruiting and activating BTK and MYD88 (*51*, *52*). Downstream of TLR engagement, BTK-dependent NF-κB activation results in the production of pro-inflammatory cytokines and chemokines (*53*), a “cytokine storm” that could increase the recruitment of monocytes/macrophages and neutrophils during the late phase of severe COVID-19 infection. BTK inhibitors such as acalabrutinib block TLR-dependent NF-κB activation in macrophages (*20*, *21*), thereby dampening the production of pro-inflammatory mediators, as occurs in an influenza-induced lung injury model (*27*). During severe COVID-19, the heightened levels of IL-1β in several COVID-19 patients (*11*, *12*) indicates the formation of an NLRP3 inflammasome that converts pro-IL-1β to mature IL-1β (*54*). BTK binds to and phosphorylates NLRP3, thereby promoting its oligomerization and assembly into an inflammasome (*24*–*26*). BTK inhibitors such as acalabrutinib inhibit inflammasome-mediated production of IL-1β, as observed in a model of influenza-induced lung injury (*27*). SARS-CoV-2, severe acute respiratory syndrome coronavirus 2; COVID-19, coronavirus disease 2019; ACE2, angiotensin-converting enzyme 2; TLR, Toll-like receptor; MyD88, myeloid differentiation primary response 88; BTK, Bruton tyrosine kinase; NF-κB, nuclear factor kappa B; NLRP3, NLR family pyrin domain containing 3; ASC, Apoptosis-associated speck-like protein containing a caspase recruitment domain; ORF3a, open reading frame 3a; IFN-γ, interferon gamma; IL, interleukin; IL-12R, IL-12 receptor; CCL2, C-C motif chemokine ligand 2; CXCL1, C-X-C motif chemokine ligand 1; CXCR2, C-X-C motif chemokine receptor 2.

We became aware of the role of Bruton tyrosine kinase (BTK) in human innate immune responses from our studies of the BTK inhibitor ibrutinib in lymphoma, in which some patients developed invasive aspergillosis during treatment (*14*). Moreover, we demonstrated that BTK-deficient mice are unable to control infection with this fungus, which is normally kept in check by monocytes/macrophages and neutrophils (*14*–*16*). While this is an uncommon complication, it raised the possibility that BTK inhibitors may modulate human inflammatory responses dominated by macrophages, as is the case in COVID-19 (*17*, *18*) and in a mouse model of this infection (Fig. 1) (*19*). In macrophages, Toll-like receptors (TLRs) recognize single-stranded RNA from viruses such as SARS-CoV-2 and initiate signaling through BTK-dependent activation of NF-κB, triggering the production of multiple inflammatory cytokines and chemokines as well as phagocytosis (Fig. 1) (*20*–*23*). In addition, BTK plays a key role in the activation of the NLRP3 inflammasome, resulting in maturation and secretion of IL-1β (*24*–*26*). Moreover, in a mouse influenza model, BTK inhibition decreased inflammatory mediators and rescued mice from lethal acute lung injury, suggesting that it may mitigate virally-induced lung damage driven by excessive inflammation (*27*).

Based on these considerations, we hypothesized that dysregulated BTK-dependent macrophage signaling is central to the exaggerated inflammatory responses and pulmonary sequelae of infection with SARS-CoV-2 and potentially other single-stranded RNA viruses. In an effort to reduce inflammation and improve clinical outcome of patients with severe COVID-19, we administered acalabrutinib, a highly specific covalent inhibitor of BTK approved in the United States for the treatment of lymphoid malignancies (*28*). Herein, we present a prospective off-label clinical study of 19 hospitalized patients with COVID-19 and severe hypoxia who also had evidence of inflammation and/or severe lymphopenia.

## RESULTS

### Patient Characteristics

This prospective off-label clinical study includes 19 hospitalized patients with severe COVID-19 who received off-label acalabrutinib between March 20, 2020 (date of treatment of the first patient) through April 10, 2020 with formal data collection completed on April 23, 2020 (Table S1). Entry criteria for this study were confirmed COVID-19 requiring hospitalization for hypoxemia (room air blood oxygen saturation (SpO2) of 94% or less) and evidence of inflammation (C-reactive protein (CRP) > 10 mg/dL and/or ferritin > 500 ng/mL) and/or lymphopenia (absolute lymphocyte count (ALC) < 1000 cells/μL). Among these patients, 13 (68%) were men and the median age was 61 years with a range of 45 to 84 years (Table 1). Eleven (58%) patients were receiving supplemental oxygen for a median of 2 days (range: 1-12), 7/11 (64%) of whom were on high flow nasal cannula at the time they began acalabrutinib (“supplemental oxygen cohort”). All but one patient had an increasing oxygen demand at the time of treatment initiation. In addition, 8 (42%) patients were receiving invasive mechanical ventilation for a median of 1.5 (range: 1-22) days prior to acalabrutinib administration (“mechanical ventilation cohort”). Coexisting medical conditions included hypertension in 16/19 (84%), obesity (body mass index > 30 kg/m^2^) in 13/19 (68%), and diabetes mellitus in 7/19 (37%) with a median (range) of 2 (0-5) co-morbid conditions per patient.

**Table 1:** Characteristics of the Patients. Obesity is defined as body mass index ≥ 30 kg/m^2^, morbid obesity is defined as body mass index ≥ 40 kg/m^2^.

	**All patients** **(N=19)**	**Supplemental Oxygen** **(N=11)**	**Mechanical Ventilation** **(N=8)**
Male sex – no. of patients (%)	13 (68%)	6 (55%)	7 (88%)
Age - median (range) – years	61 (45-84)	62 (48-84)	61 (45-77)
**Ethnicity**			
White	9 (47%)	6 (55%)	3 (38%)
Hispanic	5 (26%)	2 (18%)	3 (38%)
Black	3 (16%)	2 (18%)	1 (13%)
Asian	1 (5%)	0	1 (13%)
Middle Eastern	1 (5%)	1 (9%)	0
**Days of symptoms – median (range)**	7 (2-21)	9 (2-21)	7 (3-21)
Days of treatment – median (range)	10 (4-14)	10 (4-14)	10 (6-14)
Required intubation after treatment – no. of patients (%)		2 (18%)	
Days on ventilator before treatment – median (range)			1.5 (1-22)
Extubated after treatment – no. of patients (%)			4 (50%)
**Co-morbid conditions**			
Hypertension	16 (84%)	9 (82%)	7 (88%)
Obesity	13 (68%)	8 (73%)	5 (63%)
Morbid obesity	5 (26%)	3 (27%)	2 (25%)
Diabetes mellitus	7 (37%)	3 (27%)	4 (50%)
Obstructive sleep apnea	3 (16%)	2 (18%)	1 (13%)
Chronic kidney disease	2 (11%)	1 (9%)	1 (13%)
Coronary artery disease	1 (5%)	1 (9%)	0
Chronic obstructive lung disease	1 (5%)	1 (9%)	0
Asthma	1 (5%)	1 (9%)	0
Sarcoidosis	1 (5%)	0	1 (13%)
Rheumatoid arthritis	1 (5%)	1 (9%)	0
Chronic lymphocytic leukemia	1 (5%)	1 (9%)	0
Prostate cancer	1 (5%)	1 (9%)	0
**Signs and symptoms**			
Cough	15 (79%)	8 (73%)	7 (88%)
Dyspnea	14 (74%)	10 (91%)	4 (50%)
Fever	12 (63%	7 (64%)	5 (63%)
Myalgias/muscle weakness	6 (32%)	3 (27%)	3 (38%)
Diarrhea	2 (11%)	0	2 (25%)
Vomiting	1 (5%)	0	1 (13%)
Sore throat	1 (5%)	1 (9%)	0
Loss of taste	1 (5%)	1 (9%)	0
**Laboratory values**			
Absolute lymphocyte count (cells/μL)			
≤ 1000	15 (83%)	8 (80%)	7 (88%)
> 1000	3 (17%)	2 (20%)	1 (12%)
*Not applicable	1	1	
C-reactive protein (mg/dL)			
≥ 10	15 (79%)	7 (64%)	8 (100%)
> 3 and < 10	4 (21%)	4 (36%)	
< 3	0		
Serum ferritin (ng/mL)			
≥ 500	16 (84%)	8 (73%)	8 (100%)
< 500	3 (16%)	3 (27%)	
Fibrinogen (mg/dL)			
≥ 400	10 (100%)	6 (100%)	4 (100%)
< 400	0		
Unknown	9	5	4
D-dimer (mcg/mL)			
≥ 0.5	15 (88%)	8 (80%)	7 (100%)
< 0.5	2 (12%)	2 (20%)	
Unknown	2	1	1
IL-6 levels (pg/mL)			
≥ 15	9 (100%)	6 (100%)	3 (100%)
< 15	0		
Unknown	10	5	5

*One patient had pre-existing chronic lymphocytic leukemia making the lymphocyte count uninterpretable.

In the supplemental oxygen cohort, concomitant drugs for the treatment of COVID-19 included steroids and/or hydroxychloroquine in 5/11 (45%) patients each, and in the mechanical ventilation cohort, 6/8 (75%) and 3/8 (38%) patients, respectively, received these drugs. No patients received an anti-IL-6 receptor monoclonal antibody or remdesivir.

Laboratory evidence of inflammation with elevated CRP and/or ferritin was present in 18/19 (95%) patients with significantly elevated baseline laboratory abnormalities prior to acalabrutinib dosing including elevated CRP (> 10 mg/dL) in 15/19 (79%) patients [median (range) of 18.7 (2-31.5)]; ferritin (> 500 ng/mL) in 16/19 (84%) patients [median (range) 1240 (155-4168)]; fibrinogen (> 400 mg/dl) in 10/10 (100%) patients [median (range) 605 (409- >1000)]; D-dimer (> 0.5 mcg/mL) in 15/17 (88%) patients [median (range) 1.65 (0.48- >20)]; IL-6 (≥ 15 pg/mL) in 9/9 (100%) patients [median (range) 44 (25-89.8)]; and severely decreased ALC (≤ 1000 cells/μL) in 15/18 (83%) patients [median (range) 675 (250-1700)] (Table 1). A patient with untreated chronic lymphocytic leukemia (Patient 11) was excluded from the analysis of lymphocytes.

### Oxygen Requirements During Treatment

**Fig. 2:**
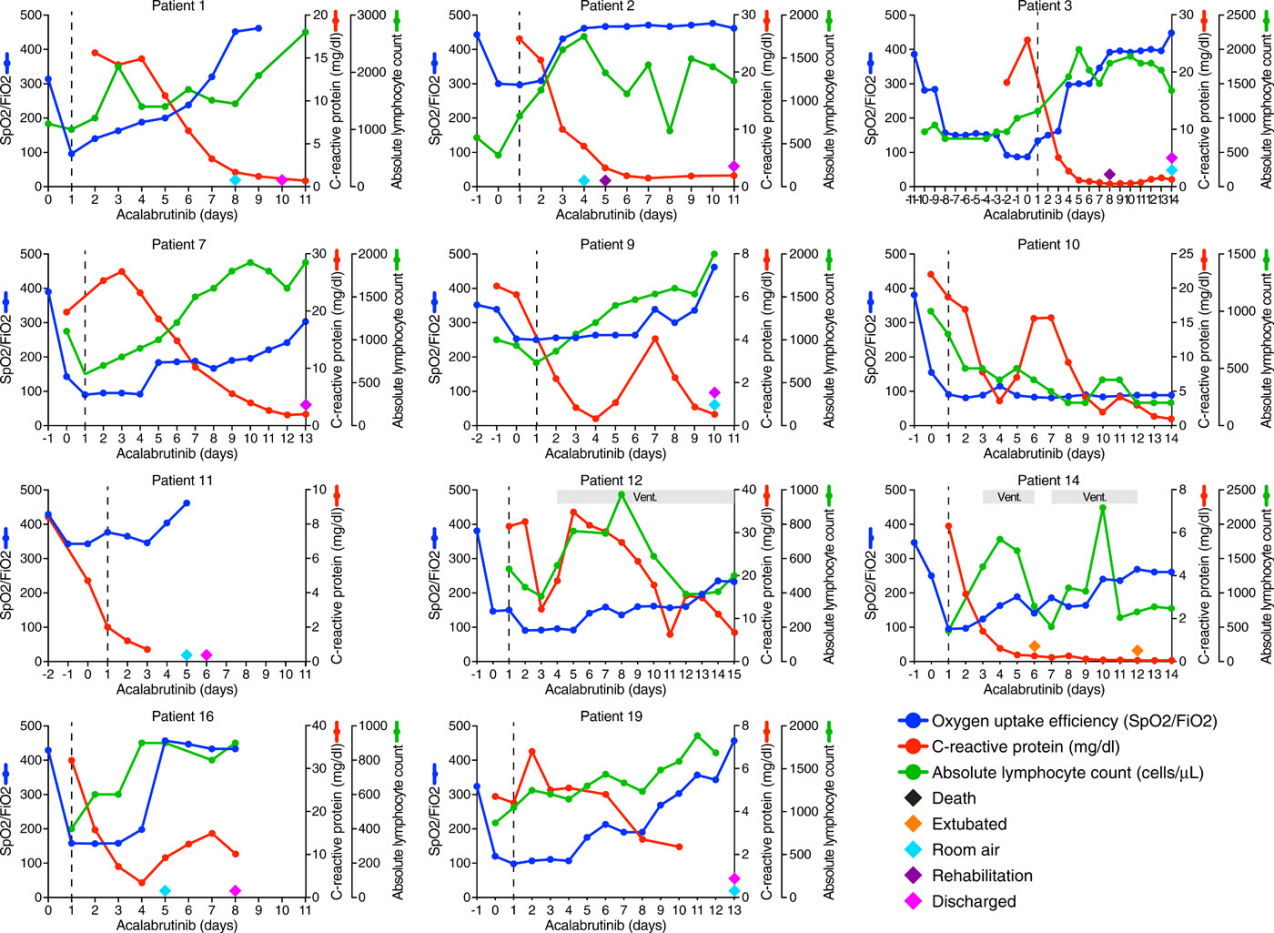
Clinical course and changes in inflammatory markers during acalabrutinib treatment in patients treated prior to intubation. Shown are measures of oxygen uptake requirement SpO2/FiO2 (% blood oxygen saturation (SpO2)/fraction of delivered oxygen (FiO2)), a ratio that accounts for both oxygen delivery and uptake (theoretical maximum 476 for 100% oxygen saturation on room air). Also shown are measures of inflammation (C-reactive protein mg/dL) and absolute lymphocyte count (cells/μL) at all available timepoints before and after acalabrutinib treatment, which was started on day 1 (dotted line). Notable clinical parameters are shown as indicated (extubation, breathing on room air, transfer to rehabilitation, hospital discharge, death). The duration of mechanical ventilation (Vent.) is indicated.

**Fig. 3:**
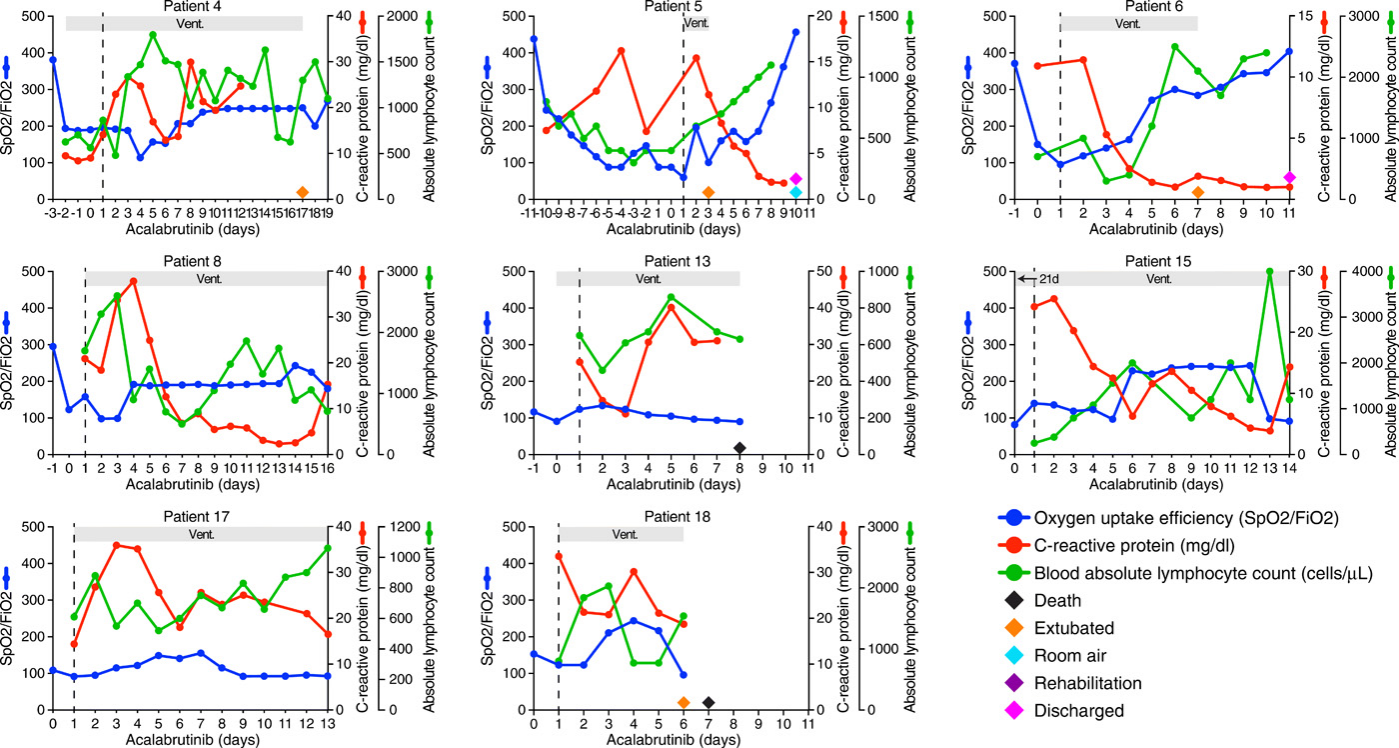
Clinical course and changes in inflammatory markers during acalabrutinib treatment in patients treated while on mechanical ventilation. See legend for Fig. 2.

To provide an estimate of a patient’s oxygen requirement, given different supplemental oxygen flow rates and concentrations, we computed the ratio of the percent blood oxygen saturation to the concentration of delivered oxygen (SpO2/FiO2) with higher values representing an improved oxygen uptake efficiency (Fig. 2, 3). The oxygen delivery rate, method of administration, delivered oxygen concentration (FiO2) and oxygen saturation values (SpO2/FiO2) are provided in Tables S2, S3.

Among 11 patients in the supplemental oxygen cohort, the median duration of follow-up from the initiation of acalabrutinib treatment was 12 (range 10-14) days. All but one patient received at least 10 days of acalabrutinib, which was the anticipated treatment duration. At the time of formal data collection, 8 (73%) patients no longer required supplemental oxygen and had been discharged from the hospital. Among 3 patients still requiring oxygen, one was on 4L/min by nasal cannula and one was on a ventilator, both with decreasing oxygen requirements, and one patient was on continuous positive airway pressure (CPAP) with a stable oxygen requirement (Fig. 2).

Eight patients on invasive mechanical ventilation were followed for a median of 12 days (range 7-30) from the initiation of acalabrutinib treatment and received the anticipated treatment duration of 10 to 14 days, with the exception of 2 patients who died (Fig. 3). In this patient group, 4/8 (50%) were extubated, two of whom were discharged, one was on 4 L/min of oxygen and weaning, and one died of an acute pulmonary embolism. Four patients remained intubated and included three patients with oscillating oxygen requirements, and one patient who died after withdrawal of support. Two of these ventilated patients had organ dysfunction due to sepsis and renal failure.

While these results are based on our prespecified cutoff for full data analysis of April 23, 2020, we obtained an outcome update as of May 28, 2020 to assess if any patients had a disease recurrence off of acalabrutinib. In the supplementary oxygen cohort, 9 patients had been discharged on room air and remained clinically well, one was still hospitalized, and one died. In the mechanical ventilation cohort, 3 patients were discharged on room air and remained well, one was discharged to rehabilitation, and 4 patients died. In all, 12 patients achieved normal oxygenation on room air (2 more that at our formal data cutoff) and none have had a recurrence.

### Laboratory Studies of Inflammation and Lymphopenia During Treatment

Laboratory measures of inflammation were monitored during treatment (Fig. 2 and 3; Tables S4, S5). The earliest and most consistent indicator of decreased inflammation was the CRP level. In the 11 patients on supplemental oxygen, CRP returned to normal in 10 (91%) patients and was decreasing in one (9%) patient (Fig. 2, 3). Available serial IL-6 levels showed normalization in 3/5 (60%) patients, and a 3-fold and 13-fold reduction from the peak value in 2 additional patients. Changes in D-dimer and fibrinogen were variable over the treatment course and did not show a clear pattern. Similarly, ferritin was quite variable and oscillated over the treatment period. In 10 patients in whom ALC was evaluable, 7 (70%) patients had increased levels on acalabrutinib, with normalization in 6, and 3 (30%) patients had decreased levels at the last available measurement (Tables S6, S7).

The 8 patients who began acalabrutinib while on mechanical ventilation showed a more variable and blunted change in laboratory values compared to those on supplemental oxygen. The CRP normalized in 2 (25%) patients, both of whom were extubated, and decreased in 3 (37%) other patients (Table S5). Three patients showed oscillating levels of CRP, one of whom was extubated and two of whom had multiorgan failure. Serial IL-6 levels available in 2 patients oscillated, and both patients had intercurrent infections. ALC values improved in a majority of these patients, with normalized values in 5/8 (63%) and oscillating values in 3/8 (37%) (Table S7).

### Correlation of CRP, ALC and Oxygen Uptake Efficiency

**Fig. 4:**
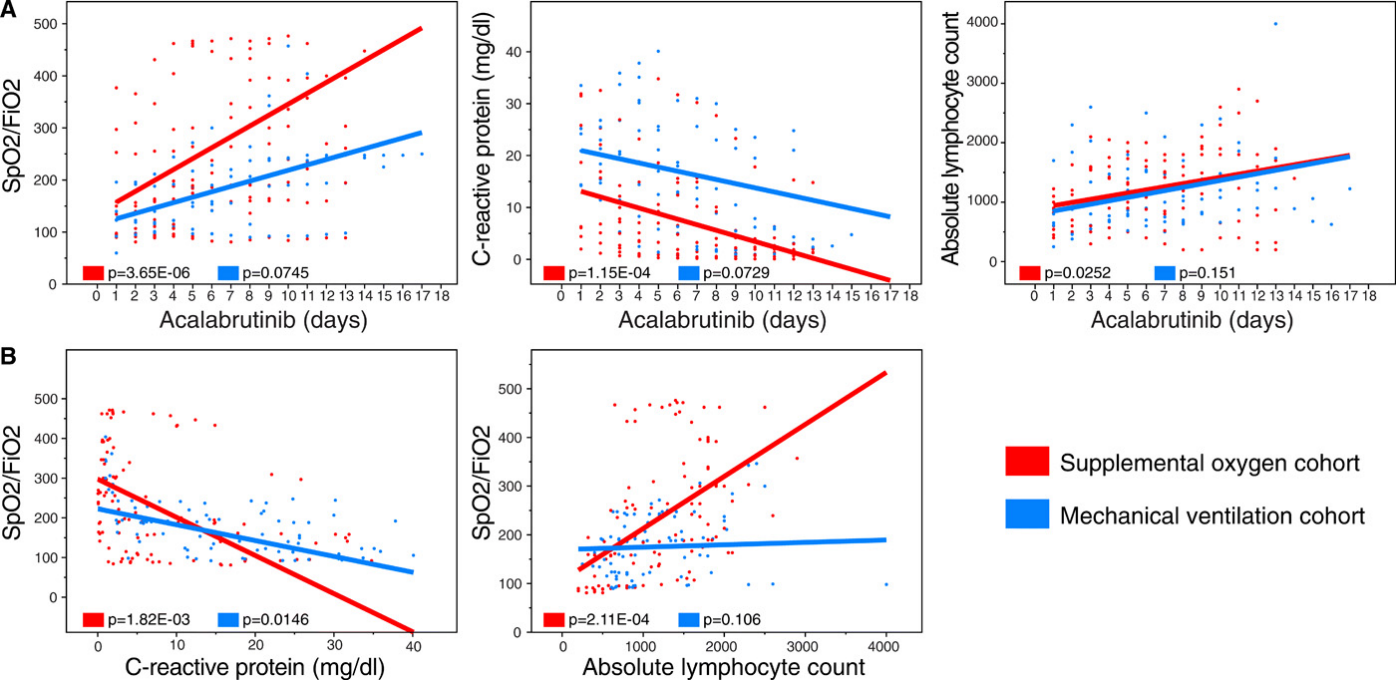
Associations between measures of pulmonary function and inflammation following acalabrutinib treatment. A. Plots of oxygen uptake efficiency (SpO2/FiO2), CRP and ALC levels versus days of acalabrutinib treatment for all patients at all time points. Patients in the supplemental oxygen and mechanical ventilation cohorts are indicated in red and blue, respectively. The trend lines shown represent the regression from a linear mixed-effect model blocked by patient. The reported p-values test the null hypothesis that the trend line has zero slope. **B.** Plots of oxygen uptake efficiency (SpO2/FiO2) versus either CRP or ALC. Trend lines and p-values as above.

Mixed-effect regression analysis showed that over time, patients in the supplemental oxygen cohort generally increased their oxygen uptake efficiency (p= 3.65E-6) and ALC (p= 0.0252) and decreased their CRP levels (p=1.15E-4) (Table S8), as illustrated by the trend lines in Fig. 4A. Moreover, CRP levels were inversely associated with ALC values in the supplemental oxygen cohort (p=5.53E-3). Similar trends were observed in the mechanical ventilation cohort but did not achieve statistical significance. CRP levels were inversely associated with oxygen uptake efficiency in both the supplemental oxygen cohort (p=1.82E-3) and in the mechanical ventilation cohort (p=1.46E-2) (Fig. 4B). ALC was directly associated with oxygen uptake efficiency in the supplemental oxygen cohort (p=2.11E-4), but not in the mechanical ventilation cohort (Fig. 4B). These results demonstrate a relatively consistent association of the CRP and ALC biomarkers with clinical improvement as measured by oxygen uptake efficiency, particularly in the supplemental oxygen cohort.

### Safety

No treatment emergent toxicities attributable to acalabrutinib were observed. Toxicities of special interest associated with acalabrutinib including cardiac arrhythmias, grade 3 or higher bleeding, diarrhea, and opportunistic infections were not observed during the treatment period (Table S9).

### BTK Activation and IL-6 Production in Monocytes from COVID-19 Patients

**Fig. 5:**
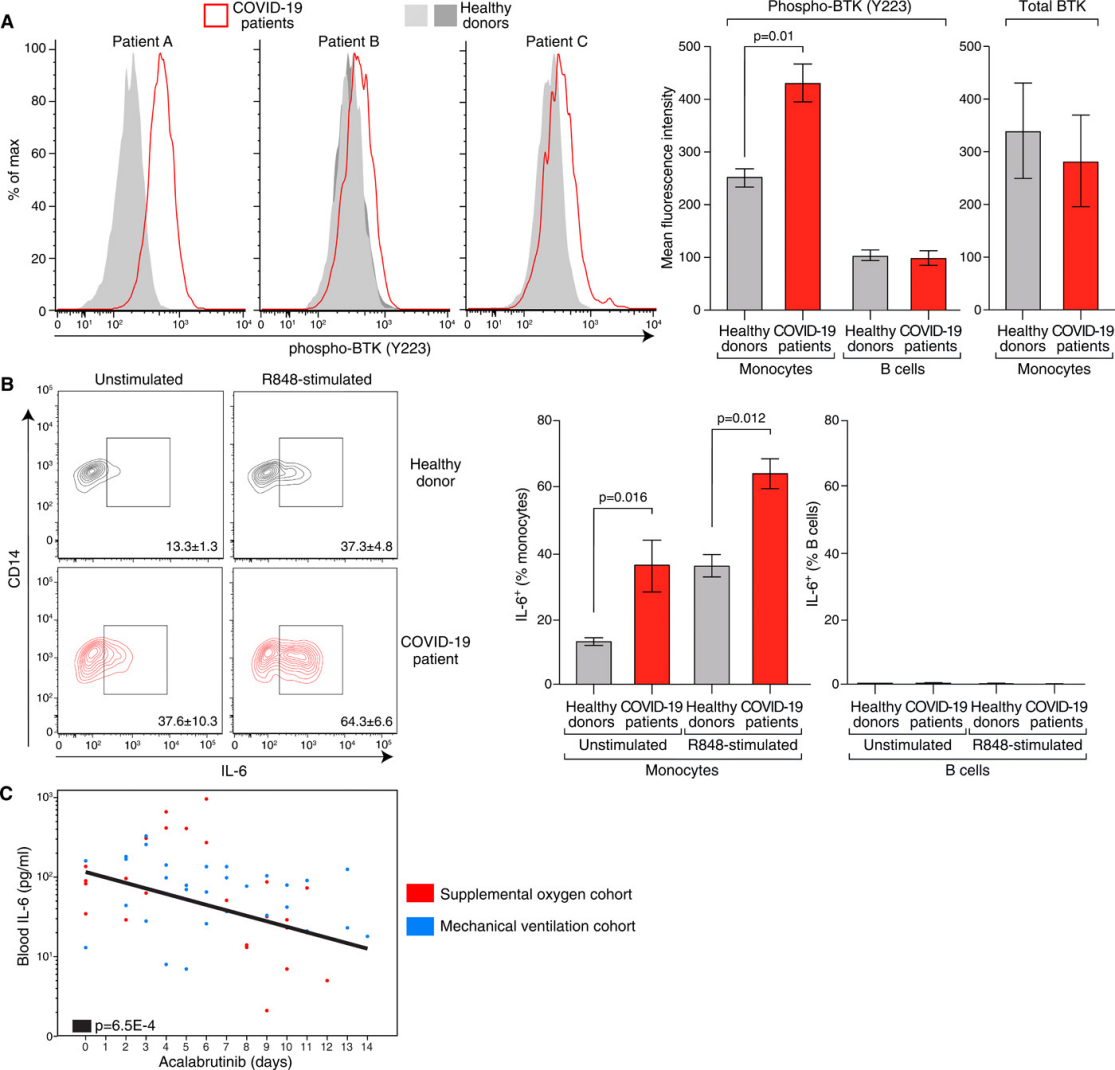
BTK activation and IL-6 production in COVID-19. A. Left panels: histograms of BTK phosphorylation in CD14^+^ blood monocytes from 3 patients with severe COVID-19 (A, B, C; Table S10) and 4 healthy volunteers, as indicated. Right panels: Summary data showing significant increase in mean fluorescence intensity of phosphorylated BTK (residue Y223) in CD14^+^ monocytes from 3 COVID-19 patients compared with 5 healthy volunteers, with no evident BTK phosphorylation in CD19^+^ B cells. Total BTK levels in blood monocytes shown in the far right panel were comparable in 3 COVID-19 patients and 5 healthy volunteers. **B.** Left panels: Representative contour plots of intracellular IL-6 production in CD14^+^ monocytes from a patient with severe COVID-19 (Patient C; Table S10) and a healthy volunteer, either as unstimulated ex vivo cells or following R848 (10 μM) stimulation, as indicated. Right panels: Summary data showing significant increase in the percent of IL-6^+^ CD14^+^ monocytes from 4 COVID-19 patients (A, B, C, D; Table S10) compared with 5 healthy volunteers, before and after R848 stimulation, with no evident IL-6 production by CD19^+^ B cells. **C.** Plot of blood IL-6 concentrations (pg/ml) on a log scale versus days of acalabrutinib treatment for patients in whom there were at least two IL-6 measurements during the plotted time course. Patients in the supplemental oxygen (n=5) and mechanical ventilation (n=3) cohorts are indicated in red and blue, respectively. The trend line shown represents the regression from a linear mixed-effect model blocked by patient for the combined cohorts due to limited data in each group. The reported p-value tests the null hypothesis that the trend line has zero slope. All quantitative data in 5A and 5B represent means ± SEM.

To examine whether the target of acalabrutinib, BTK, was activated in patients with COVID-19, we studied BTK autophosphorylation at residue Y223 in whole blood samples from 3 patients with severe COVID-19 (Table S10) and in 5 healthy volunteers (Fig. S1). We observed a significantly increased mean fluorescence intensity of phosphorylated BTK in CD14+ monocytes from patients with severe COVID-19 relative to that observed in healthy volunteers, an increase that was not due to differential levels of total BTK (Fig. 5A). We next examined expression of IL-6 protein by immune cells in the blood of COVID-19 patient since the production of this cytokine is known to be increased by BTK activity in normal human monocytes/macrophages (Fig. S2). Flow cytometric analysis of unstimulated whole blood samples revealed a significant increase in the percentage of IL-6^+^ CD14^+^ monocytes in patients with severe COVID-19 (n=4) compared with healthy volunteers (n=5; Fig. 5B). Treatment of these whole blood samples with the small molecule R848, a mimic of TLR7 and TLR8 activation by single strand RNA, increased the percentage of IL-6^+^ blood monocytes, with significantly higher levels in samples from COVID-19 patients compared to healthy controls (Fig. 5B). Of note, the percentage of IL-6^+^ monocytes in patients with severe COVID-19 without ex-vivo restimulation was comparable to that observed in monocytes from healthy volunteers following R848 stimulation (Fig. 5B). BTK phosphorylation and IL-6 production were not observed in B cells in the same whole blood samples, demonstrating that BTK was specifically activated in monocytes from COVID-19 patients. In keeping with these findings, blood IL-6 levels in COVID-19 patients on our clinical study decreased during acalabrutinib treatment (p=6.5E-4) (Fig. 5C).

## DISCUSSION

Our clinical and correlative laboratory studies have revealed that BTK is a likely instigator of the pathological inflammatory response in severe COVID-19. In accordance with World Health Organizations guidance (*29*), we prospectively administered acalabrutinib off-label with therapeutic intent to 19 hospitalized patients with severe COVID-19, based on the known role of BTK in innate immune cells. All but one patient had increasing oxygen requirements at the time of treatment initiation, and all but 4 patients were on high-flow oxygen or invasive mechanical ventilation, indicating the severity of the disease in this series. The oxygenation and clinical status of most patients on supplemental oxygen improved relatively rapidly following acalabrutinib initiation, which was temporally associated with a normalization of inflammatory markers. Although the patients on mechanical ventilation had a more variable clinical response to acalabrutinib, improved oxygenation in half of these patients allowed them to be extubated. Our laboratory studies of ex vivo blood samples from patients hospitalized with COVID-19 revealed significantly elevated BTK phosphorylation in peripheral blood monocytes compared with healthy volunteers, demonstrating that the target of acalabrutinib is activated in these innate immune cells. This finding supports our view that the apparent beneficial effect of acalabrutinib in these patients was due to on-target inhibition of BTK. More generally, this study highlights the opportunity to improve outcomes in severe COVID-19 by modulating the host inflammatory response.

While most patients infected with SARS-CoV-2 have a limited disease not requiring hospitalization, the patients in this series had progressed to a hyperinflammatory phase of this infection that can be fatal and for which there are no proven treatment strategies. All patients in our series had elevated inflammatory markers including CRP, ferritin and/or IL-6 (*2*, *30*, *31*). The majority of patients also had increased D-dimer levels, which can be associated with a coagulopathy that is common in COVID-19. Many patients in this series had a severely depressed ALC, which has also been associated with severe COVID-19. Acalabrutinib administration was temporally associated with a change in several of these biomarkers of inflammation suggesting that BTK activation was triggering this pathology. In the majority of patients, levels of the inflammatory marker CRP normalized or decreased substantially, as did IL-6 levels. Likewise, lymphopenia rapidly normalized in most patients, possibly related to decreased inflammatory cytokines or chemokines (*30*–*32*). A link between improved pulmonary function and decreased inflammation was strongly suggested by the inverse relationship between a measure of oxygen uptake efficiency (SpO2/FiO2) and CRP levels.

The apparent beneficial effect of acalabrutinib was clearly different between patients who were on supplemental oxygen and those who required mechanical ventilation. In the supplemental oxygen cohort, oxygenation improved in 82%, with 73% discharged on room air despite high preexisting oxygen requirements in most. Though the benefit of acalabrutinib was less dramatic in patients on ventilators, half were extubated after receiving acalabrutinib. The association between oxygen uptake efficiency and normalization of CRP was also evident in the mechanical ventilation cohort. These patients were quite heterogeneous clinically, including patients who had major organ dysfunction such as renal failure or who had been ventilated for an extended period prior to acalabrutinib administration. Though we expected that the optimal time to initiate anti-inflammatory treatment would be prior to deterioration requiring intubation, these results suggest that BTK inhibition may provide significant benefit to a subset of patients with COVID-19 on ventilators. Further correlative studies will be needed to understand the basis for response or resistance to BTK inhibition in patients with such advanced disease.

Since our study investigated the effect of a limited course of acalabrutinib in severe COVID-19, we were interested in whether the disease recurred after acalabrutinib cessation. Among 12 patients who achieved room air status on acalabrutinib, none have had a recurrence suggesting that a short course of acalabrutinib was sufficient to quell the disease clinically.

While all patients fulfilled pre-specified treatment characteristics, limited patient numbers and the absence of a control group may lead to an inaccurate estimate of treatment efficacy and safety. The safety of any drug is always of paramount concern but is further heightened when used in an untested disease state such as severe COVID-19 in which multi-organ dysfunction occurs. It is within this context that we administered acalabrutinib with careful consideration of risks and potential benefits. The safety profile of acalabrutinib is well defined in the context of long-term use over months to years in patients with chronic lymphocytic lymphoma (*33*). Acalabrutinib has greater kinase selectivity than other clinically available BTK inhibitors, which likely contributes to its favorable safety profile (*34*). The most common adverse events associated with long-term acalabrutinib therapy include low-grade headache, diarrhea, pyrexia and upper respiratory tract infections with rare grade 3 or 4 toxicity (*33*). While ibrutinib has been associated with major hemorrhage and atrial fibrillation, these side effects occur rarely in patients treated with acalabrutinib. Inhibition of the innate immune system by BTK inhibitors has been associated with a small increase in opportunistic infections, particularly in the setting of combination chemotherapy or high dose corticosteroids and/or long-term use (*14*, *35*). It is notable that we did not observe any of the above toxicities attributable to acalabrutinib treatment, suggesting that in the context of COVID-19, acalabrutinib is relatively well tolerated. However, since we have only treated a small cohort of patients, the safety profile of acalabrutinib in patients with severe COVID-19 needs to be confirmed in a prospective clinical trial.

Ex vivo analysis of blood samples from patients with severe COVID-19 revealed BTK activation in monocytes in all cases, as evidenced by significantly increased BTK phosphorylation compared with monocytes from healthy volunteers. Notably, blood B cells did not have evidence of BTK activation, suggesting that monocytes/macrophages may be the relevant in vivo target of acalabrutinib in COVID-19. Consistent with this hypothesis, IL-6 production was elevated in monocytes from COVID-19 patients while there was no evidence of IL-6 production in B cells. Notably, BTK was apparently active in the entire population of blood monocytes, given the shift of the entire histogram of BTK phosphorylation to higher levels. This finding is less likely attributable to trafficking of a subpopulation of activated monocytes from the lung to the blood but more consistent with systemic activation of BTK in monocytes, either by the virus, viral RNA, or another circulating inflammatory mediator. This pervasive activation of BTK in monocytes/macrophages argues that the clinical benefit of acalabrutinib stemmed from its ability to turn off pathological BTK signaling in innate immune cells, which in turn extinguished the hyperinflammatory process in these patients.

Acalabrutinib may have been effective because it targets a source of cytokine production in innate immune cells rather than the downstream effector functions of individual cytokines. Other therapeutic strategies have been considered for COVID-19, including corticosteroids. These agents provided little or no benefit in previous coronavirus epidemics and are not recommended for COVID-19 (*36*). Despite the absence of documented benefit, more than half of the patients in our series received steroid support. Hydroxychloroquine was administered to 42% of our patients despite also having no proven benefit in severe COVID-19. Other immunomodulatory strategies have been proposed, such as monoclonal antibodies targeting the IL-6 or IL-1 receptors, which were not administered to patients in our series (*11*). Since multiple inflammatory cytokines and chemokines are elevated in patients with COVID-19 (*12*) inhibition of any one inflammatory mediator may only partially reduce the inflammatory process. While BTK inhibitors interfere with B cell activation and could potentially lower anti-viral antibody titers, this concern may be mitigated by the timing of administration to patients with severe COVID-19, who are typically hospitalized 7 or more days following initial infection. A more complete understanding how BTK inhibitors modulate the immune pathophysiology of COVID-19 will require the use of preclinical model systems in concert with detailed immune profiling of patients with COVID-19, before and during treatment with a BTK inhibitor.

After we initiated our prospective off-label clinical study of acalabrutinib in COVID-19, investigators interested in the role of BTK in COVID-19 reported that among 6 patients with confirmed COVID-19 who were taking the BTK inhibitor ibrutinib chronically for their hematologic malignancy, only one patient was hospitalized (*37*). In the reported median age of 66 years for these patients, however, the Centers for Disease Control and Prevention (CDC) reported a hospitalization rate of 12.2%, which is in fact lower that the hospitalization rate in this small series (16.6%) (*38*). Furthermore, since the authors provide no information on comorbidities that are associated with severe COVID-19, it is impossible to conclude that ibrutinib ameliorated the disease course. In one hospitalized patient who was already on ibrutinib, the authors reported a clinical improvement following an increase in the ibrutinib dose, possibly representing a salutary effect or alternatively attributable to other factors in this clinically complicated case. Mechanistically, the authors proposed that ibrutinib inhibited TLR-mediated signaling and pulmonary inflammation by targeting hematopoietic cell kinase (HCK), but acalabrutinib has no significant inhibitory activity against HCK (*34*), arguing against its relevance.

If BTK inhibition is of clinical benefit in severe COVID-19, as is supported by our data, it raises the question of which BTK inhibitor would be optimal in this clinical setting given the association of COVID-19 with arrythmias and other serious systemic sequelae of the inflammatory process. Acalabrutinib, unlike ibrutinib, has no detectable inhibitory activity against the immunologically important kinase ITK or against EGFR, a key signaling receptor in epithelial cells. While the efficacies of acalabrutinib and ibrutinib are comparable in hematological malignancies, the BTK selectivity of acalabrutinib may reduce unwanted clinical toxicities. In this regard, ibrutinib has a higher incidence of serious bleeding and pro-arrhythmic side effects than acalabrutinib, toxicities that may worsen the outcome of patients with severe COVID-19 (*39*).

The clinical and laboratory findings in patients with severe COVID-19 are indicative of macrophage activation syndrome (*40*), which occurs in diverse clinical settings and is characterized by elevated CRP, IL-6 and other inflammatory cytokines, suggesting that the immunopathology of severe COVID-19 involves dysregulation of macrophage homeostasis. Consistent with this hypothesis, post-mortem examination of COVID-19 lungs revealed an increased preponderance of monocyte/macrophage cells in pulmonary alveoli (*17*, *18*). BTK activation occurs in macrophages when TLRs bind single-stranded RNA, as may occur in SARS-CoV-2 infection, leading to NF-κB-dependent expression of multiple inflammatory cytokines and chemokines, including IL-6 which we observed was induced in COVID-19 monocytes and decreased in plasma following acalabrutinib treatment (Fig. 1). BTK also regulates the formation of NLRP3 inflammasomes in macrophages by physically associating with NLRP3 and phosphorylating its linker domain, triggering oligomerization and formation of inflammasomes (*24*–*26*). BTK inhibition, either genetically or pharmacologically, markedly attenuates inflammasome formation in response to diverse stimuli (*24*). Although we have focused our model on macrophages, BTK is also known to control signaling in neutrophils (*41*), megakaryocytes (*42*), and platelets (*43*), which may also contribute to the immunopathology of severe COVID-19 and be kept in check by BTK inhibitors.

Several co-morbidities that are associated with severe COVID-19 (*44*) – obesity, hypertension, atherosclerosis and type 2 diabetes – have been linked individually and as part of the metabolic syndrome to a heightened inflammatory state characterized by inflammasome activation in macrophages (*45*, *46*). These comorbidities could conceivably establish a heightened inflammatory “set point” that affects how macrophages respond to SARS-CoV-2 infections. This concept has been variously called “trained immunity” or “innate immune memory” and results from epigenetic changes in gene expression in response to disease states or infections (*47*). Since infectious agents are powerful modifiers of innate immune memory (*47*), it will be important to gauge whether SARS-CoV-2 infection exacerbates comorbid disease states and whether BTK inhibitors can prevent this.

This prospective study of patients with severe COVID-19 highlights the potential benefit of BTK inhibition and has led to a confirmatory international prospective randomized controlled clinical trial. Given the activation of BTK and production of IL-6 that we detected in COVID-19 monocytes, we propose that BTK inhibitors target pathological monocyte/macrophage activation and dampen the cytokine storm, which consequently may improve outcomes in these patients. More broadly, our findings raise the prospect that the morbidity of other disease states associated with macrophage activation, including severe influenza infections (*27*), may similarly depend on BTK function, supporting clinical trial evaluation of BTK inhibitors in these clinical settings as well.

## MATERIALS AND METHODS

### Patient Selection

We developed a list of selection criteria to identify patients who would potentially benefit from the off-label use of acalabrutinib to block the excessive host inflammatory response and improve clinical outcome. Patients at high risk for toxicity from acalabrutinib including a known history of fungal infections, bleeding disorders, recent hemorrhagic stroke, ventricular arrythmias, malabsorption syndromes, or patients who required strong CYP3A4 inhibitors were not considered for acalabrutinib. The selection criteria included hospitalized patients with confirmed COVID-19 and hypoxia (room air blood oxygen saturation (SpO2) of 94% or less) requiring supplemental oxygen and ferritin ≥ 500 ng/mL, C-reactive protein ≥ 10 mg/dL and/or an absolute lymphocyte count < 1000 cells/μL. Patients were ≥ 18 years, capable of swallowing pills or had an enteric feeding tube and were not pregnant or breast feeding. We communicated with physicians at five hospitals to identify hospitalized patients who met these criteria and had individual case-based discussions with the treating physicians regarding the use of acalabrutinib as an off-label treatment for patients who were either deteriorating or not improving on best supportive care.

### Acalabrutinib Treatment

Patients received the approved acalabrutinib dose of 100 mg orally or per enteric feeding tube twice daily for 10 days (patients on supplemental oxygen) and 14 days (patients on mechanical ventilation). We recommended that acalabrutinib be discontinued in patients who developed significant drug-related toxicity, which was not observed. Guidance was provided regarding the safe preparation of an acalabrutinib solution for patients who required an enteric feeding tube (see Supplementary Materials).

Supportive care was up to the treating physicians but with the following guidance: avoid the use of concomitant corticosteroids, including inhaled steroids based on the observation that the use of steroids with BTK inhibitors may slightly increase the risk of Aspergillus infections (*14*). Furthermore, anecdotal reports suggest that corticosteroids may adversely affect COVID-19 (*36*). Patients who are receiving corticosteroids for COVID-19 at the time of acalabrutinib institution should be weaned off as appropriate. Avoid the use of proton pump inhibitors (PPI) and substitute H2 blockers if possible to reduce adverse effects on drug absorption. Patients receiving a strong CYP3A4 inhibitor should be switched to an alternative medication as medically indicated to reduce their effects on drug clearance. Off-label use of hydroxychloroquine may increase the risk of cardiac toxicity (*48*).

### Study Assessments and Monitoring

Local institutional practice guidelines were followed regarding indications for supplemental oxygen delivery, need for mechanical ventilation, and laboratory studies of complete blood counts with differential cell counts, and full chemistry panels. We used the oxygen saturation/fraction of inspired oxygen ratio (SFR) to monitor daily changes in the patient’s oxygenation status (*49*). To monitor for signs of inflammation, we recommended, where possible, frequent monitoring of CRP, ferritin, fibrinogen, D-dimer, and IL-6 levels, which are non-experimental tests. All other studies were as per the local physicians.

### Ethical Considerations

The study involved the off-label administration of an FDA-approved drug in the setting of a pandemic for which there were no known effective treatments. Because this study was not conducted under an approved protocol, consultation with local institutional review boards by each individual hospital was undertaken to ensure that use of off-label acalabrutinib was ethically justified for the clinical situation. The local institutional review board and/or the appropriate clinical leadership of each institution approved the use of clinical data in this report. Ethical guidance for the use of off-label drugs during a global pandemic is provided by a World Health Organization document that addresses the use of unproven interventions during infectious disease outbreaks (*29*). In the context of an outbreak characterized by high mortality, it is considered ethical to offer patients experimental interventions on an emergency basis and outside of clinical trials provided: 1. there are no proven effective treatments; 2. it is not possible to initiate clinical studies immediately; 3. preliminary data exists to support a drug’s off-label use; 4. the risk-benefit ratio for the patient is favorable; 5. a qualified scientific advisory committee has approved the drug’s use; 6. the patient’s informed consent is obtained; and 7. the treatment results are documented and shared with the scientific community in a timely manner. The present report adheres to these guidelines. Patients and normal volunteers who participated in the correlative component of this report were enrolled on a National Institutes of Health approved clinical protocol (NCT00001467; NCT01200953).

### Informed Consent

Each patient or their legally authorized representative underwent oral informed consent by a physician experienced with acalabrutinib at each hospital, which included a discussion of treatment risk and benefit, and was documented in the medical record. We explained that the off-label use of acalabrutinib to block the excessive host inflammatory response in viral pneumonia had not been tested in clinical trials, was only of theoretical benefit, and potential benefits and safety in this setting were unknown. We also discussed the clinical experience with acalabrutinib and its known safety profile. The treating physician was included in these discussions to inform on other treatment options for severe COVID-19. On a case-by-case basis, we explained the risks/benefits to the patient or their legally authorized representative in order to make them aware of all potential treatment alternatives during their severe COVID-19 illness. We explained that the risk of adverse events associated with 10 to 14 days of treatment was low, but included the possibility of increased secondary infections, new onset of cardiac arrhythmias, increased risk of bleeding, and gastrointestinal disturbances such as diarrhea or worsening liver test abnormalities. Patients and normal volunteers who participated in the correlative study were enrolled on a National Institutes of Health approved clinical protocol (NCT00001467; NCT01200953) and provided written informed consent in accordance with the Declaration of Helsinki.

### Correlative Study Participants

Four patients who were hospitalized with severe COVID-19 at the NIH Clinical Center (n = 2) or George Washington University Hospital (n = 2) enrolled in NIH IRB-approved protocols (NCT00001467; NCT01200953). All four patients had confirmed COVID-19 by PCR testing, were hypoxemic (SpO2 < 94% on room air) with bilateral pulmonary infiltrates on imaging, and increased CRP levels (Table S10). Five healthy volunteers enrolled in a NIH IRB-approved protocol (NCT01386437). Each COVID-19 patient was tested on a different day with 1 or 2 different healthy volunteers each time. Patient and accompanying healthy volunteer blood samples were harvested in the morning and were processed for flow cytometry-based analyses of BTK phosphorylation and IL-6 within 2-3 hours of blood harvesting as described below. All study participants provided written informed consent in accordance with the Declaration of Helsinki.

### Analysis of Phosphorylated BTK in Whole Blood Using Flow Cytometry

Heparinized whole blood was aliquoted in round-bottom polystyrene tubes (Corning) to which live/dead fixable blue stain (Thermo Fisher) and mouse antibodies against human CD14 (clone M5E2; Biolegend) and human CD19 (clone HIB19; Biolegend) were added, mixed and incubated at 37°C for 20 min. Subsequently, the cells in whole blood were fixed by adding 2 mL pre-warmed Phosflow Lyse/Fix buffer (BD Biosciences) for 10 min in a water bath at 37°C with intermittent mixing. The fixed cells were then centrifuged at 2000 rpm for 6 min, Lyse/Fix buffer was removed, and the cells were washed with ice-cold PBS. The fixed cells were then permeabilized using 100% ice-cold methanol (Thermo Fisher). After 30 min of incubation in methanol, the cells were washed once with ice-cold PBS. After two additional washes with PBS containing 0.5% BSA (Thermo Fisher) and 0.01% NaN_3_, cells were incubated overnight at 4°C with a rabbit antibody against human BTK phosphorylated at Y223 (clone EP420Y; Abcam) and mouse antibody against total human BTK (clone 53; BD Biosciences), along with mouse IgG (Thermo Fisher) to minimize non-specific binding. The next morning, cells were washed twice with FACS buffer (PBS containing 0.5% BSA and 0.01% NaN_3_) and stained using an Alexa Fluor® 488-conjugated goat anti-rabbit IgG secondary antibody (Abcam, cat# ab150077) along with mouse antibodies against human HLA-DR (clone G46.6; BD Biosciences) and CD16 (clone 3G8; Biolegend). Mouse IgG (Thermo Fisher) was added to minimize non-specific binding. After 30 min of incubation on ice, the cells were washed twice with FACS buffer, resuspended in FACS buffer and analyzed using a 5-laser LSRFortessa flow cytometer (BD Biosciences). The data were exported and analyzed using FlowJo (TreeStar). CD14^+^ monocytes were defined as live, single, HLA-DR^+^ CD14^+^ cells and B cells were defined as live, single, HLA-DR^+^ CD19^+^ cells.

### Analysis of Intracellular IL-6 in Whole Blood Using Flow Cytometry

Heparinized whole blood was aliquoted in round-bottom polystyrene tubes (Corning) and brefeldin A (10 μg/mL; BD Biosciences), monensin (2 μM; Biolegend) and antibodies against human CD3 (clone SK7; Thermo Fisher) and CD4 (clone RPA-T4; BD Biosciences) were added. The blood was either left unstimulated or was stimulated with R848 (10 μM; Invivogen). After 4h of incubation at 37°C in a humidified incubator containing 5% CO_2_, cells in whole blood were washed with PBS and stained using antibodies against CD123 (clone 6H6; Thermo Fisher), CD56 (clone NCAM16.2; BD Biosciences), CD8 (clone SK1; Biolegend), HLA-DR (clone G46-6; BD Biosciences), CD19 (clone HIB19; Biolegend), CD14 (clone MøP9; BD Biosciences) and CD16 (clone 3G8; BD Biosciences). Mouse IgG (Thermo Fisher) was added along with staining antibodies to minimize non-specific binding. After incubation with the staining antibodies, cells were washed with PBS and stained using live/dead fixable blue stain (Thermo Fisher). Subsequently, the cells were washed with PBS containing 1% fetal bovine serum (R&D Systems) and were fixed with paraformaldehyde (2% w/v; Thermo Fisher). Fixed cells were then washed with FACS buffer (PBS containing 0.5% BSA and 0.01% NaN_3_) and stored overnight at 4°C. The next morning, cells were permeabilized using FACS buffer containing saponin (Sigma). Permeabilized cells were then stained for intracellular IL-6 using mouse anti-human IL-6 antibody (clone MQ2-13A5; Biolegend), in the presence of mouse IgG to minimize non-specific binding. Staining was carried out in FACS buffer containing saponin, after which the cells were washed, resuspended in FACS buffer and analyzed using a 5-laser LSRFortessa flow cytometer (BD Biosciences). The data were exported and analyzed using FlowJo (TreeStar). CD14^+^ monocytes were defined as live, single, HLA-DR^+^ CD14^+^ cells and B cells were defined as live, single, HLA-DR^+^ CD19^+^ cells.

Comparison of the frequency of IL-6^+^ CD14^+^ monocytes or B cells under unstimulated or stimulated conditions and of the mean fluorescence intensity (MFI) of phosphorylated BTK in CD14^+^ monocytes or B cells between patients with severe COVID-19 and healthy volunteers were performed using an unpaired *t* test or Mann-Whitney test where appropriate, using GraphPad Prism 8.0 and were presented as means ± SEM.

### Statistical Analyses

The statistical associations among time, SpO2/FiO2, CRP, ALC and log IL-6 concentration were modeled as a linear mixed-effect regression, with the time points for each patient treated as independent observations, and with the intercept and slope estimates being blocked by patient. The model was fit using the lmer function from the lme4 R-package (*50*). P-values were calculated via a Wald test and are two-sided.

Comparisons of the frequency of IL-6^+^ CD14^+^ monocytes or B cells under unstimulated or stimulated conditions and of the mean fluorescence intensity (MFI) of phosphorylated BTK in CD14^++^monocytes or B cells between patients with severe COVID-19 and healthy volunteers were performed using an unpaired *t*-test or Mann-Whitney test where appropriate, using GraphPad Prism 8.0 and were presented as means ± SEM.

## SUPPLEMENTARY MATERIALS

immunology.sciencemag.org/cgi/content/full/5/48/eabd0110/DC1 Supplementary Methods (instructions on preparation of acalabrutinib for an enteric feeding tube) Figure S1. Gating strategy for flow cytometric analysis of phosphorylated and total BTK. Figure S2. Gating strategy for flow cytometric analysis of IL-6. Table S1. Treatment centers. Table S2. Clinical course of supplemental oxygen cohort. Table S3. Clinical course of mechanical ventilation cohort. Table S4. Laboratory tests for inflammatory markers during acalabrutinib treatment in supplemental oxygen cohort. Table S5. Laboratory tests for inflammatory markers during acalabrutinib treatment in mechanical ventilation cohort. Table S6. Other laboratory tests during acalabrutinib treatment in supplemental oxygen cohort. Table S7. Other laboratory tests during acalabrutinib treatment in mechanical ventilation cohort. Table S8. Statistical analysis of laboratory changes related to oxygenation status. Table S9. Adverse events of special interest during treatment with acalabrutinib. Table S10. Characteristics of COVID-19 patients who underwent flow cytometry-based immunological analyses. Table S11. Raw data file (Excel spreadsheet).
